# Functional Impact of the *N*-terminal Arm of Proline Dehydrogenase from *Thermus thermophilus*

**DOI:** 10.3390/molecules23010184

**Published:** 2018-01-16

**Authors:** Mieke M. E. Huijbers, Ilona van Alen, Jenny W. Wu, Arjan Barendregt, Albert J. R. Heck, Willem J. H. van Berkel

**Affiliations:** 1 Laboratory of Biochemistry, Wageningen University & Research, Stippeneng 4, 6708 WE Wageningen, The Netherlands; mieke.huijbers@wur.nl (M.M.E.H.); ilona.vanalen@wur.nl (I.v.A.); jenny.wu@wur.nl (J.W.W.); 2 Biomolecular Mass Spectrometry and Proteomics, Bijvoet Center for Biomolecular Research and Utrecht Institute of Pharmaceutical Sciences, Utrecht University, Padualaan 8, 3584 Utrecht, The Netherlands; a.barendregt@uu.nl (A.B.); a.j.r.heck@uu.nl (A.J.R.H.); 3 Netherlands Proteomics Center, Padualaan 8, 3584 Utrecht, The Netherlands

**Keywords:** flavoprotein, proline dehydrogenase, protein engineering, protein oligomerization, solubility tag, suicide inhibition, TIM-barrel

## Abstract

Proline dehydrogenase (ProDH) is a ubiquitous flavoenzyme that catalyzes the oxidation of proline to Δ^1^-pyrroline-5-carboxylate. *Thermus thermophilus* ProDH (TtProDH) contains in addition to its flavin-binding domain an *N*-terminal arm, consisting of helices αA, αB, and αC. Here, we report the biochemical properties of the helical arm truncated TtProDH variants ΔA, ΔAB, and ΔABC, produced with maltose-binding protein as solubility tag. All three truncated variants show similar spectral properties as TtProDH, indicative of a conserved flavin-binding pocket. ΔA and ΔAB are highly active tetramers that rapidly react with the suicide inhibitor *N*-propargylglycine. Removal of the entire *N*-terminal arm (ΔABC) results in barely active dimers that are incapable of forming a flavin adduct with *N*-propargylglycine. Characterization of V32D, Y35F, and V36D variants of ΔAB established that a hydrophobic patch between helix αC and helix α8 is critical for TtProDH catalysis and tetramer stabilization.

## 1. Introduction

**Scheme 1:**
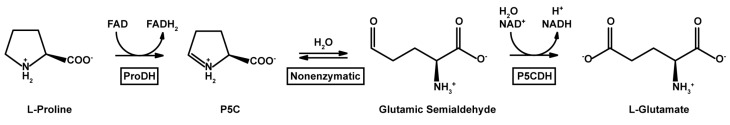
Conversion of l-proline to l-glutamate by proline dehydrogenase (ProDH) and Δ^1^-pyrroline-5-carboxylate dehydrogenase (P5CDH). Scheme based on [10].

Proline dehydrogenase (ProDH; EC 1.5.5.2) is a ubiquitous enzyme involved in proline catabolism. ProDH catalyzes the flavin-dependent oxidation of l-proline to Δ^1^-pyrroline-5-carboxylate (P5C). After P5C hydrolysis, the resulting glutamic semialdehyde (GSA) is oxidized to glutamate through the action of Δ^1^-pyrroline-5-carboxylate dehydrogenase (P5CDH; EC 1.2.1.88) (Scheme 1). ProDH and P5CDH exist as separate monofunctional enzymes in eukaryotes and some bacteria, but are fused into a bifunctional [1,2] or trifunctional [3] enzyme in other bacteria. In these multi-functional enzymes, called proline utilization A (PutA), the *C*-terminus of ProDH is fused to P5CDH, allowing for the channeling of the P5C/GSA intermediate between the enzymes [4,5,6,7,8,9].

**Figure 1:**
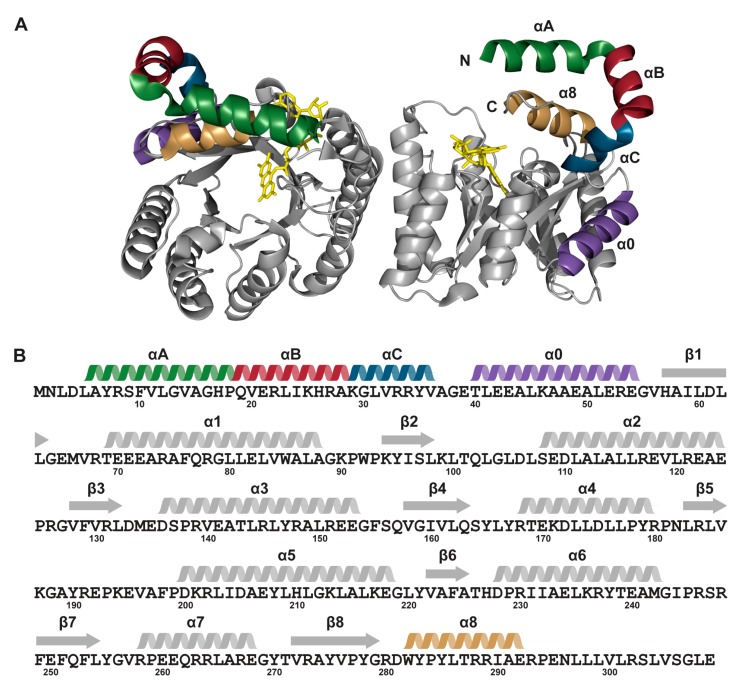
Structural features of *Thermus thermophilus* proline dehydrogenase (TtProDH). (**A**) Three-dimensional model of the crystal structure of the TtProDH dimer (PDB entry 2G37). The *N*-terminal helices αA (green), αB (red), and αC (blue) are indicated, as well as helix α0 (purple) and the *C*-terminal helix α8 (brown); (**B**) Amino acid sequence of TtProDH. Secondary structural elements on top of the sequence have the same colors as in Figure 1A. Figure 1A is based on [19].

ProDH has a distorted (βα)_8_ TIM-barrel fold (Figure 1A), which is conserved throughout the PutA/ProDH family [11,12]. Opposed to the classic TIM-barrel fold, the ProDH barrel begins with a helix (α0) rather than a strand (Figure 1). This extra helix occupies the location that is normally reserved for α8. As a consequence, α8 is not located alongside β8, but on top of the barrel [1,13,14]. The distorted location of α8 is crucial for catalysis, since it contributes three strictly conserved residues (Tyr-x-x-Arg-Arg) that interact with the substrate [13,14].

The *N*-terminal sequence of ProDH is poorly conserved. Monofunctional eukaryotic ProDHs, including the human enzyme [15], have an elongated *N*-terminus when compared to monofunctional bacterial ProDHs [11,12]. ProDH from *Thermus thermophilus* (TtProDH) contains an *N*-terminal arm consisting of three helices: αA, αB, and αC (Figure 1). Helix α8 fits into the cleft, which is formed by helices αA, αB and αC. This cleft is assumed to be involved in channeling P5C/GSA between TtProDH and TtP5CDH [1,16].

Previously, we showed that TtProDH is overproduced in *Escherichia coli* (*E. coli*) when its *N*-terminus is fused to maltose-binding protein (MBP) [17] and that the recombinant enzyme does not discriminate between FAD and FMN as cofactor [18]. Replacing Phe10 and Leu12 in helix αA with Glu residues (further referred to as MBP-TtProDH variant EE) successfully released MBP-TtProDH from non-native self-association, yielding homogeneous tetramers [19]. Although TtProDH crystallizes as a dimer (Figure 1) [1], it is worthy of note that in solution, the enzyme remains a tetramer after removal of the MBP solubility tag [13].

To investigate the functional impact of the *N*-terminal arm of TtProDH in catalysis and oligomerization in closer detail, we constructed MBP-fused variants, lacking, respectively, one (ΔA), two (ΔAB), or three (ΔABC) *N*-terminal helices. ΔA and ΔAB turned out to be highly active tetramers, while ΔABC was an almost inactive dimer. To further probe the role of helix αC, we addressed the properties of site-specific variants V32D, Y35F, and V36D of ΔAB. This revealed that a hydrophobic patch between helix αC and helix α8 is critical for TtProDH catalysis and tetramer stabilization.

## 2. Results

### 2.1. Protein Expression and Purification

**Figure 2:**
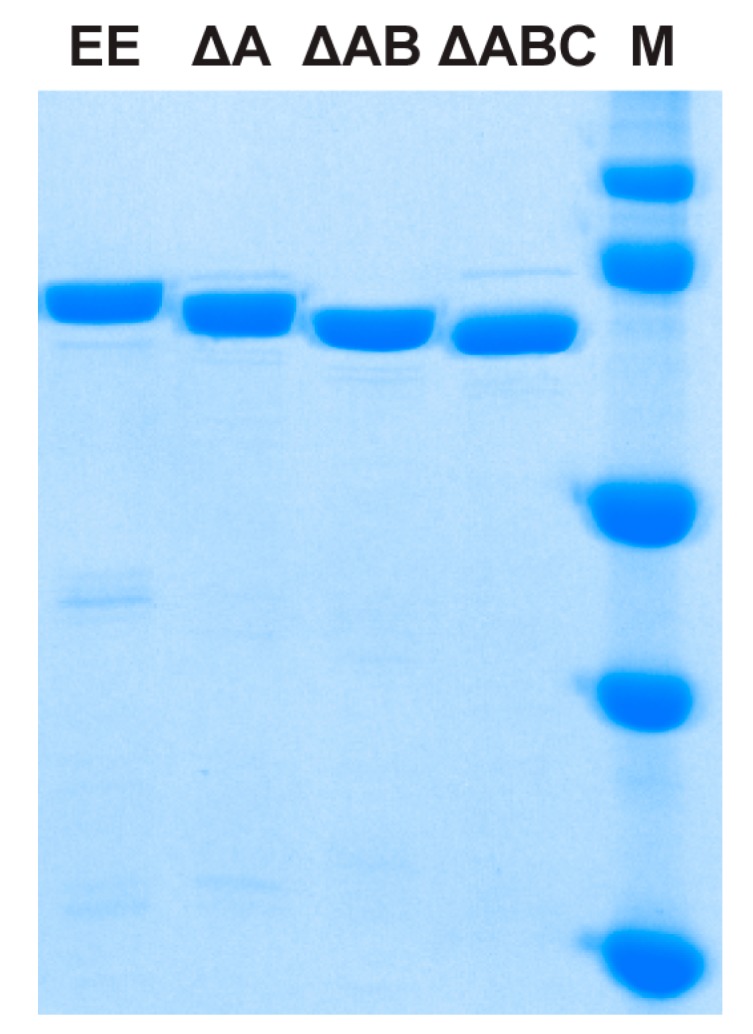
Purified MBP-TtProDH variants visualized on sodium dodecyl sulfate polyacrylamide gel electrophoresis (SDS-PAGE). Molecular masses of marker proteins (M), from top to bottom: 100, 75, 50, 37, 25 kDa.

MBP-TtProDH EE, ΔA, ΔAB and ΔABC were overproduced in *E. coli* TOP10 cells. From 1 L of culture about 200–250 mg of each variant was purified, yields that we have described before for the heterologous production of MBP-TtProDH [17,19]. From SDS-PAGE analysis of the purified enzymes, it can be appreciated that sequential removal of three helices results in a gradual decrease of subunit molecular mass (Figure 2).

Previously, we demonstrated that removal of the MBP fusion tag with trypsin does not significantly affect the catalytic properties and oligomerization behavior of TtProDH [17]. However, as found with EE [19], trypsinolysis of the arm-truncated variants did not result in homogeneous preparations. Therefore, we used the MBP-fused variants for further studies.

### 2.2. Spectral Properties

**Figure 3:**
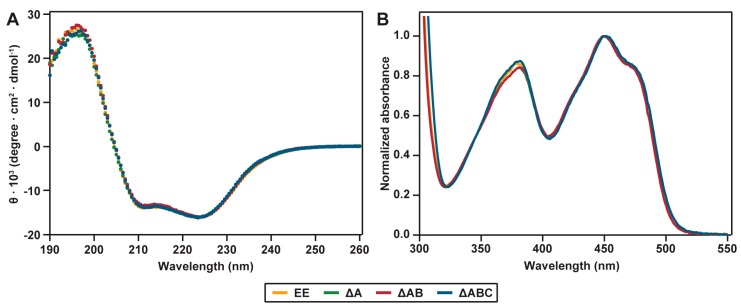
Spectral properties of EE, ΔA, ΔAB, and ΔABC. (**A**) Far-UV CD spectra; (**B**) Visible flavin absorption spectra.

The far-UV CD spectra of EE and the *N*-terminal variants are very similar (Figure 3A) and comparable to that of MBP-TtProDH [17]. The visible flavin absorption spectra of EE and the arm-truncated variants (Figure 3B) are also nearly identical to that of MBP-TtProDH [17,19], except that the lowest energy absorption band of ΔABC has shifted about 2 nm to higher wavelengths. These data support that there are no major structural changes and that deletion of the *N*-terminal helices does not significantly alter the microenvironment of the flavin-binding site. A recent crystallographic study confirmed that ΔABC (residues 38–279; PDB 5M42) has a similar overall structure as TtProDH (PDB 2G37) with an rmsd value of 0.338 Å (for 221 Cα atoms of A chains) [1,18].

### 2.3. Hydrodynamic Properties

**Figure 4:**
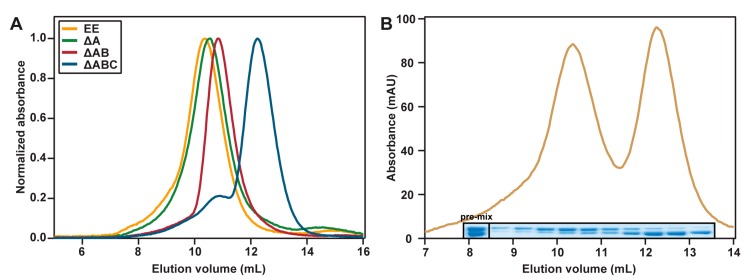
Hydrodynamic properties of EE, ΔA, ΔAB, and ΔABC as monitored by Superdex 200 size-exclusion chromatography. (**A**) Elution pattern of the MBP-TtProDH variants; (**B**) Hydrodynamic properties of EE and ΔABC, mixed in equal amounts and equilibrated at room temperature overnight. SDS-PAGE of the gel filtration fractions is also shown.

EE purifies as a tetramer [17,19]. The truncated variants ΔA and ΔAB also form tetramers as indicated by size exclusion chromatography (Figure 4A). ΔABC elutes mainly as a dimer (Figure 4A), suggesting that αC plays a role in the tetramerization process. Previously, we showed that TtProDH prepared from MBP-TtProDH also forms tetramers, but that the addition of 1 M GuHCl induces the formation of dimers [17]. The crystal structure also shows a dimeric structure (cf. Figure 1A).

Mixing of EE and ΔABC followed by analytical gel filtration reveals two separate peaks, a peak for the tetrameric species of EE and a peak for the dimeric species of ΔABC (Figure 4B). This indicates that mixing of the two enzyme forms does not lead to EE-ΔABC association. Moreover, SDS-PAGE of the gel filtration fractions shows that no subunit exchange occurs (inset Figure 4B).

Additional information about the oligomeric state of the different variants was obtained by native mass spectrometry. The estimated masses (Table 1) confirm that ΔA and ΔAB exist predominantly as tetramers, and ΔABC as dimer. With ΔABC, minor amounts of tetramers are present, in agreement with the analytical gel filtration results (Figure 4A). For ΔA and ΔAB, some dimers are observed with native mass spectrometry, and for ΔABC monomers, although their abundance is rather low. Denaturing the different complexes enabled an accurate mass measurement of the individual subunits (Table 1). These masses show that all of the variants exist of identical subunits.

The experimental masses of native and denatured ΔABC do not correspond with the predicted masses. The observed species appears to be *C*-terminally truncated [18]. Based on the estimated mass, cleavage occurs before Arg288, leading to the removal of a part of the *C*-terminal tail with sequence (288-RRIAERPENLLLVLRSLVSGLE-309). This truncated form has a predicted subunit mass of 71,884.9 Da, close to the measured mass of 71,899.3 Da [18].

### 2.4. Catalytic Properties

**Figure 5:**
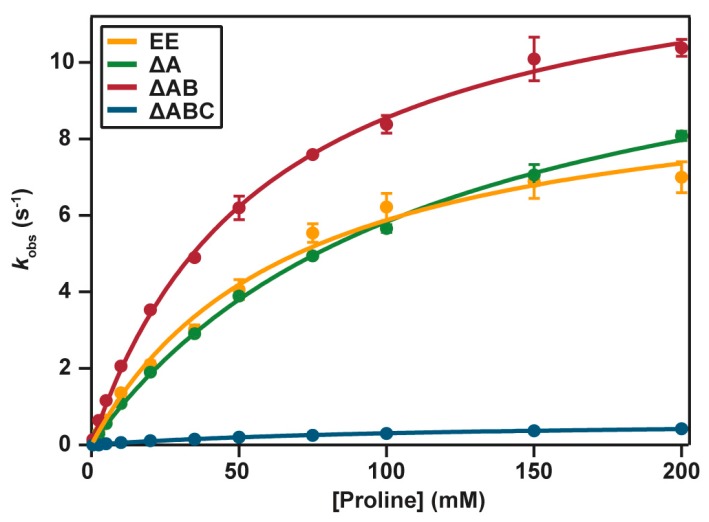
Steady-state kinetics of EE, ΔA, ΔAB, and ΔABC. The fitted curves are retrieved from non-linear regression analysis applying the Michaelis-Menten equation. Each data point was retrieved in triplicate.

Figure 5 presents an overview of the steady-state kinetic properties of the MBP-TtProDH variants. The kinetic parameters derived from these experiments are summarized in Table 2. The proline *K*_m_ values of EE, ΔA, ΔAB, and ΔABC are comparable. However, ΔA and ΔAB have a slightly higher activity than EE while ΔABC is almost inactive. These data suggest that αA and αB are not required for optimal activity, and that further deletion of helix αC is critical for catalysis.

Since TtProDH has a low but significant proline oxidase activity [1], it was of interest to address the oxygen reactivity of the *N*-terminal arm variants. For all of the variants described above, micromolar concentrations of enzyme were needed to reliable measure consumption of oxygen, and very low specific activities were observed (Table 2). Nevertheless, these data confirm that removal of the complete *N*-terminal arm (ΔABC) also impairs the proline oxidase activity of MBP-TtProDH.

### 2.5. Reaction with N-propargylglycine

**Figure 6:**
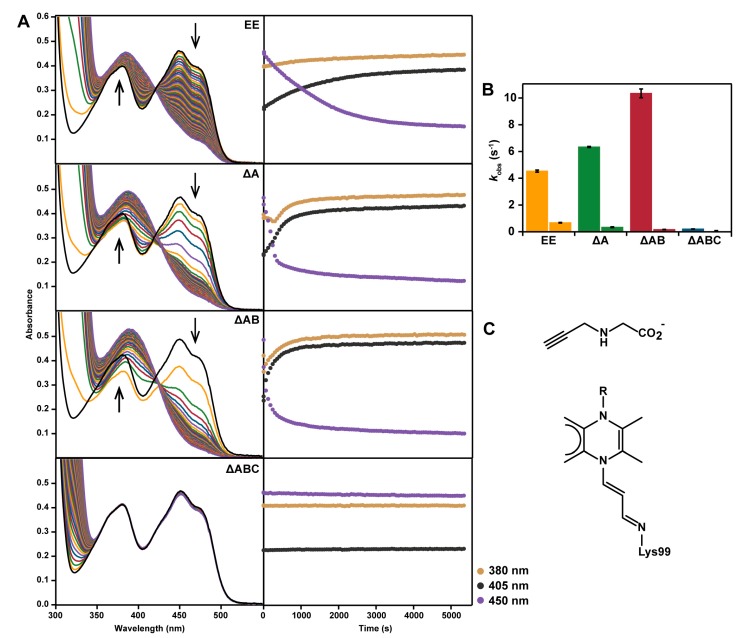
Reactivities of the *N*-terminal arm TtProDH variants with *N*-propargylglycine. (**A**) Left: absorption spectral changes of the variants upon incubation with *N*-propargylglycine. The black line indicates the spectrum before the addition of *N*-propargylglycine. Spectra were recorded at 1 min intervals for 90 min. Right: absorbance changes of the variants upon incubation with *N*-propargylglycine followed at 380, 405, and 450 nm; (**B**) Activity of the different enzyme variants before and after incubation with *N*-propargylglycine. Error bars are based on three replicates. (**C**) Chemical structure of *N*-propargylglycine and the covalent Lys99-FAD adduct.

TtProDH is irreversibly inactivated by the suicide inhibitor *N*-propargylglycine [10]. Inactivation involves the initial oxidation of *N*-propargylglycine to *N*-propargyliminoglycine and the subsequent formation of a bicovalent linkage between flavin N(5) and the ε-amino group of Lys99 (Figure 6C). Lys99 is located in the loop between β2 and α2 and is involved in binding the carboxylic moiety of proline [1]. Upon reaction with *N*-propargylglycine the absorption maximum of TtProDH at 450 nm disappears, while the maximum around 380 nm gradually increases. Furthermore, the peak at 380 nm shifts to longer wavelengths. This behavior is indicative of the initial reduction of the flavin and the subsequent formation of the covalent Lys99-FAD adduct [8,10]. After reaction with *N*-propargylglycine, the enzyme is locked in the reduced state.

To probe the catalytic features of the MBP-TtProDH variants in further detail, we investigated the reactivity of EE and the arm-truncated variants with *N*-propargylglycine. Figure 6A shows that all of the variants except ΔABC form a covalent flavin adduct, and that the reactions result in similar absorption changes, as observed before with TtProDH [10]. However, a more careful analysis of the kinetics of the reactions reveals significant differences.

For EE, ΔA, and ΔAB, flavin reduction and adduct formation are clearly observed (Figure 6A). Reduction of EE by *N*-propargylglycine is relatively slow, as evidenced from the time-dependent decrease in absorption at 450 nm. Flavin reduction is immediately followed by covalent adduct formation as evidenced from the absorbance increase around 380 and 405 nm. Variants ΔA and ΔAB reveal an increased rate of reduction compared to EE. This corresponds with the increased *k*_cat_ values for these variants (Table 2). The peak at 380 nm shows a red shift to 385 nm for EE and to 388 nm for ΔA and ΔAB. Furthermore, both for ΔA and ΔAB, the increase in absorbance around 388 nm is more pronounced.

ΔABC shows neither flavin reduction nor formation of a flavin adduct (Figure 6A). However, a slow rise in absorption with a maximum at 290 nm is observed in the near-UV region, pointing to the conversion of *N*-propargylglycine to *N*-propargyliminoglycine. Activity measurements with DCPIP suggest that *N*-propargylglycine is indeed a poor substrate for ΔABC.

Activity of the different enzyme variants before and after incubation with 2.5 mM of *N*-propargylglycine was measured (Figure 6B). For EE, residual activity after 90 min of incubation with the inhibitor is about 13%. For ΔA and ΔAB the activity loss is even more pronounced, in good agreement with the spectral results. The residual activity of ΔABC after 90 min incubation with *N*-propargylglycine is extremely low (Figure 6B).

### 2.6. Interactions between Helix αC and Helix α8

**Figure 7:**
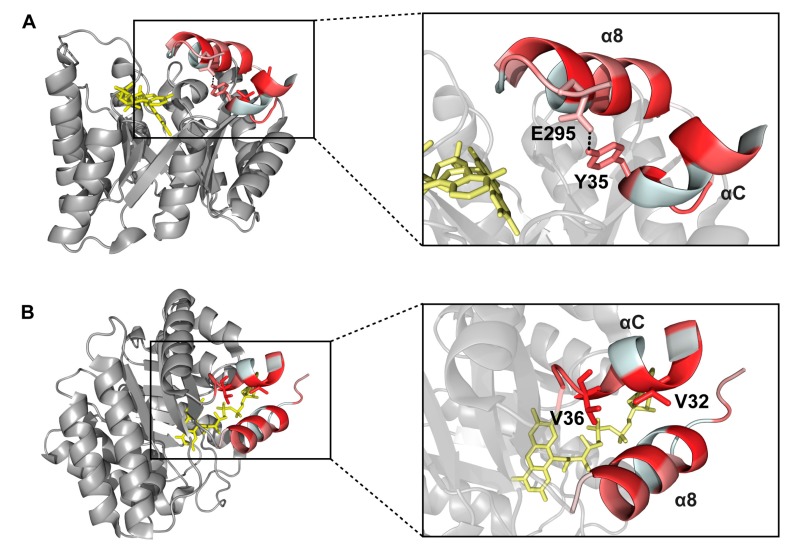
Interactions between helix αC and helix α8. The residues in helices αC and α8 are colored in a hydrophobic (red) to non-hydrophobic (white) gradient, according to the hydrophobicity scale introduced by Eisenberg et al. [20]. The remaining part of the catalytic domain is shown in grey and the FAD cofactor is shown in yellow. (**A**) Ion pair Tyr35 (αC) and Glu295 (α8); (**B**) Hydrophobic patch between αC and α8 with Val32 (αC) and Val36 (αC) indicated. Data according to the crystal structure of TtProDH (PDB entry 2G37).

ΔABC is produced as a catalytically impaired dimer. In addition, this MBP-TtProDH variant is truncated at its *C*-terminal helix α8. This suggests that helix αC is important for the stabilization of helix α8. The three-dimensional model of the crystal structure of TtProDH suggests that there is a hydrogen bond between Tyr35 of αC and Glu295 of α8 (Figure 7). Glu295 is part of the sequence that is cleaved off in ΔABC. To investigate whether the Tyr-Glu interaction is important for the stabilization of helix α8, we changed Tyr35 to Phe. In addition, there is a hydrophobic patch between helix αC and α8 (Figure 7). To examine the importance of this patch for the stabilization of helix α8, Val32, and Val36 individually were changed into Asp. The single amino acid substitutions were introduced in MBP-TtProDH ΔAB, since this variant is fully active and forms stable tetramers. In this way, we only look at the interaction between helix αC and α8 without possible interference of helix αA and αB.

**Figure 8:**
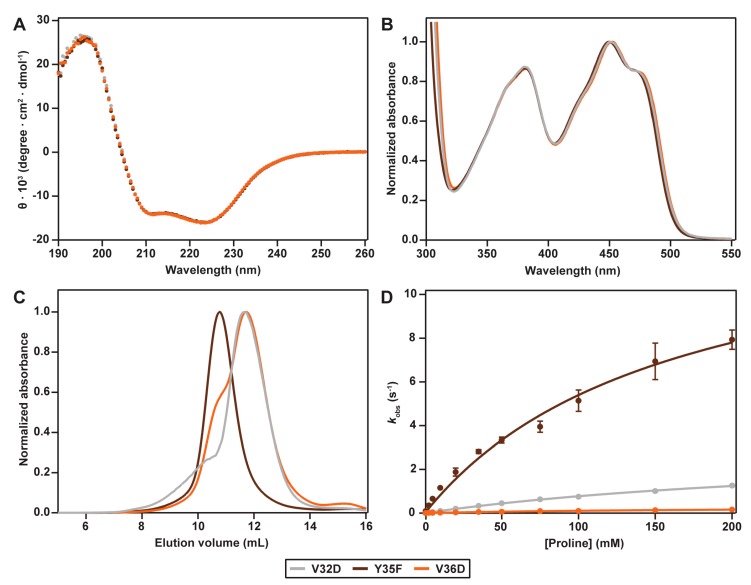
Properties of helix αC variants of ΔAB. (**A**) Far-UV CD spectra; (**B**) Visible flavin absorption spectra; (**C**) Hydrodynamic properties as monitored by Superdex 200 size-exclusion chromatography; and, (**D**) Steady-state kinetic data as determined with the Proline:DCPIP assay. Each data point was retrieved in triplicate. The raw kinetic data of the Proline:DCPIP assay, used to determine the *K*_m_ and *k*_cat_ values, is provided in Table S1.

The far-UV CD spectra of V32D, Y35F, and V36D (Figure 8A) are identical to the far-UV CD-spectra of the *N*-terminal variants (Figure 3A). The visible flavin absorption properties of the ΔAB variants show that the low energy absorption bands of V32D and V36D have shifted about 2 nm to higher wavelength, as compared to those of ΔAB and Y35F (Figure 3B and Figure 8B). Thus, the flavin absorption properties of V32D and V36D resemble those of ΔABC (Figure 3B).

Size exclusion chromatography of Y35F indicates that substitution of Tyr35 with Phe does not affect the tetrameric nature of ΔAB (Figure 8C). Therefore, the interaction between Tyr35 and Glu295 does not seem critical for stabilization of helix α8. V32D and V36D elute much later from the gel filtration column than Y35F, suggesting that these variants mainly form dimers (Figure 8C). Native MS of Y35F confirms the presence of tetramers. Furthermore, V32D and V36D are present both as tetramers and dimers (Table 1). The mass of denatured Y35F shows the presence of identical subunits, while V32D and V36D show two subunit masses, of which one corresponds with the predicted mass (Table 1). The differences between the subunit masses suggest that both V32D and V36D are partially intact and partially cleaved before R288 (predicted mass 73,001.2 Da), indicating flexibility and instability of helix α8.

Analysis of the catalytic properties of Y35F confirms that the interaction between Tyr35 and Glu295 is not crucial for the functioning of TtProDH (Figure 8D). The minor decrease in catalytic efficiency when compared to ΔAB mainly results from a slight increase in *K*_m_ for the proline substrate (Table 2). V32D and V36D, on the other hand, are poorly active (Figure 8D). With these variants, a considerable decrease in catalytic efficiency is observed (Table 2). Oxidase activity of especially V36D is very low (Table 2), as found with ΔABC.

**Figure 9:**
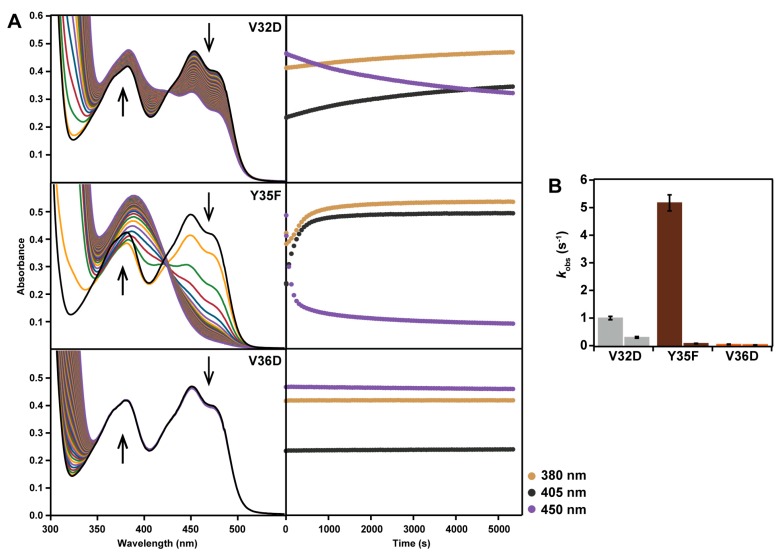
Reactivities of helix αC variants of ΔAB with *N*-propargylglycine. (**A**) Left: absorption spectral changes of the variants upon incubation with *N*-propargylglycine. The black line indicates the spectrum before the addition of *N*-propargylglycine. Spectra were recorded at 1 min intervals for 90 min. Right: absorbance changes of the variants upon incubation with *N*-propargylglycine followed at 380, 405, and 450 nm; (**B**) Activity of the different enzyme variants before and after incubation with *N*-propargylglycine. Error bars are based on three replicates.

The catalytic features of the helix αC variants were investigated in more detail by probing their reactivity with the suicide inhibitor *N*-propargylglycine (Figure 9). For Y35F (Figure 9A), the rates for flavin reduction and adduct formation are similar as found for ΔAB (Figure 6A). For V32D, flavin reduction and adduct formation are also observed (Figure 9A), but these processes are much slower than for ΔAB. The flavin prosthetic group of V36D is not reduced by *N*-propargylglycine, and the typical absorption increase around 380 nm, indicative for adduct formation, is also not observed (Figure 9A). However, as for ΔABC, a slow rise in absorption occurs in the region 300–350 nm, pointing to the conversion of *N*-propargylglycine to *N*-propargyliminoglycine.

All the helix αC variants lose activity when treated with *N*-propargylglycine. After incubation for 90 min, Y35F is almost completely inactivated, but V32D and V36D retain 30% and 63% of their original (rather low) activity (Figure 9B).

## 3. Discussion

Proline dehydrogenases contain a conserved (βα)_8_ TIM-barrel domain and an *N*-terminal arm that differs in length among monofunctional bacterial and eukaryotic ProDHs. In this study, we investigated the functional impact of the *N*-terminal arm of *Thermus thermophilus* ProDH. We analyzed variants that lack one (ΔA), two (ΔAB), or three (ΔABC) *N*-terminal helices and compared these variants to the EE variant of MBP-fused TtProDH. The latter variant is a highly active soluble form of the enzyme that exclusively forms tetramers [19]. Spectral analysis showed that truncation of the *N*-terminal arm of TtProDH does neither affect the binding of the FAD cofactor nor the microenvironment of the flavin isoalloxazine ring.

We already showed that non-native aggregation of MBP-TtProDH is due to the hydrophobicity of helix αA. Replacing Phe10 and Leu12 of αA with glutamates eliminates the formation of larger aggregates [19]. Here, we established that the complete removal of helix αA (ΔA), or removal of helices αA and αB (ΔAB), has the same effect: no aggregates are observed and ΔA and ΔAB exclusively form tetramers. This confirms that αA is responsible for the in vitro aggregation of MBP-TtProDH and that both helices αA and αB are not essential for the tetramerization process. Furthermore, estimation of kinetic parameters revealed that αA and αB are not essential for the enzymatic activity of MBP-TtProDH. Actually, ΔAB is the most active MBP-fused TtProDH variant reported thus far. Incubations with the mechanism-based inhibitor *N*-propargylglycine yielded supporting information about the catalytic competence and the structural integrity of ΔA and ΔAB. The flavin cofactor of both arm-truncated variants is rapidly reduced, immediately followed by covalent adduct formation. This leads to irreversible suicide inactivation of the enzymes by *N*-propargylglycine (Figure 6).

Helix αC turned out to be more crucial for oligomerization and catalysis. Removal of the complete *N*-terminal arm (ΔABC) resulted in catalytically impaired dimers and poor reactivity with *N*-propargylglycine. MS analysis showed that ΔABC has lost 22 residues at the *C*-terminus, including Arg288 and Arg289, involved in proline binding [1]. Thus, the dissociation of tetramers into dimers and loss in catalytic performance of ΔABC might be caused by the removal of αC and/or the partial absence of helix α8. We do not have indications that the loss in activity is caused by dimer formation, since the dimer-dimer interactions in the tetramer seem to be relatively weak. The oxidase activity remains low for all deletion variants, suggesting that the removal of the *N*-terminal helices does not improve the access of oxygen to the flavin.

For *Deinococcus radiodurans* ProDH (DrProDH, PDB 4H6Q) [21], *Bradyrhizobium japonicum* PutA (BjPutA, PDB 3HAZ) [22] and *Geobacter sulfurreducens* PutA (GsPutA, PDB 4NM9) [7], a conserved Arg-Glu ion pair (Arg288-Glu65 in TtProDH) (Figure S1) is suggested to act as an active site gate. Arg288 is present in helix α8 while Glu65 is present in the β1-α1 loop. In the presence of substrate, this loop moves towards the active site and the Arg-Glu ion pair can form. In ΔABC, helix α8 is cleaved before Arg288, thereby disrupting this ion pair and excluding this possibility for active site stabilization.

Triggered by the properties of ΔABC, we introduced single amino acid substitutions in ΔAB that might disrupt the interaction between helix αC and helix α8 in a more delicate way. Replacement of Tyr35 with Phe showed that the hydrogen bond interaction between Tyr35 and Glu259 is not crucial for the catalytic performance of the enzyme, and also not for protein tetramerization. Incubation with *N*-propargylglycine confirmed that the flavin reactivity and structural integrity of Y35F are highly comparable to that of ΔAB.

The Tyr35–Glu295 interaction is not conserved in DrProDH, although both amino acid residues are present in a region with highly conserved sequence. The latter enzyme is the only other monofunctional ProDH of which a crystal structure is available [21]. In DrProDH, Tyr35 is replaced by a phenylalanine (Phe34), while Glu295 is replaced by an arginine (Arg298). The absence of the Tyr-Glu interaction in DrProDH is another indication that this ion pair is not of great importance for the structural integrity of TtProDH.

The V32D and V36D variants of ΔAB provided further insight into the functional role of helix αC. Both of the variants mainly form dimers, show partial proteolytic processing at the *C*-terminus, and display low catalytic efficiencies. This lends support to the proposal that the hydrophobic patch between helix αC and α8 (Figure 7) is not only important for tetramer formation, but also for the proper functioning of the active site. Assessment of the reactivity of V32D and V36D with *N*-propargylglycine revealed interesting differences. While slow flavin reduction and covalent adduct formation are observed for V32D, these processes do not take place with V36D. In agreement with this, V36D is less active with proline than V32D, and resembles ΔABC in this respect (Table 2). When we compare the hydrophobicity of helices αC and α8 against their counterparts in DrProDH, we observe a similar hydrophobic patch between both helices. In addition, analysis of the crystal structures of *E. coli* PutA (PDB 4O8A), BjPutA, and GsPutA suggests that helix α8 might also be stabilized by hydrophobic contacts, although in PutA enzymes helix α8 might also be stabilized by additional *N*- and *C*-terminal helices.

In conclusion, our results strengthen the idea that helix αC is involved in TtProDH tetramerization and stabilization of helix α8. The hydrophobic patch between helix αC and helix α8 stimulates tetramerization and is important for catalysis because it orients helix α8 for proper binding of the substrate and interaction with the active site.

We have shown that helix αA and αB of TtProDH are not crucial for the in vitro activity of the enzyme. However, αA and αB of TtProDH might be of importance in vivo, by serving as a docking interface for partner enzyme TtP5CDH during channeling [16] and/or for interaction with the membrane. Concerning these issues, it is of interest to study the function of the elongated *N*-terminus of monofunctional eukaryotic ProDHs, especially of human ProDH [15].

## 4. Materials and Methods

### 4.1. Construction of MBP-TtProDH Variants

Three *N*-terminally shortened MBP-fused variants were constructed; each variant was shortened with one additional *N*-terminal helix. To amplify the DNA, pBAD-MBP-TtProDH [19] was used as template DNA. The primers listed in Table 3 were used for amplification. Using *Eco*RI and *Hind*III restriction sites, the amplified fragments were introduced into a pBAD-MBP vector, which resulted in *N*-terminal fusions of the TtProDH variants to MBP. The resulting constructs MBP-TtProDH ΔA, MBP-TtProDH ΔAB, and MBP-TtProDH ΔABC were verified by automated sequencing of both strands (Macrogen, Seoul, Korea) and the plasmids were transformed to *E. coli* TOP10 host cells for recombinant expression.

The obtained plasmid for MBP-TtProDH ΔAB was used as a template to construct point mutations in helix αC. V32D, Y35F, and V36D were constructed using the procedure described earlier [19], with the exception that in this case both a forward and a reverse primer were used (Table 3).

### 4.2. Expression and Purification of MBP-TtProDH Variants

The MBP-TtProDH variants were purified following a previously described procedure [19]. In short, the variants were produced in *E. coli* TOP10 cells and purified using amylose affinity and anion-exchange chromatography.

### 4.3. Protein Analysis

Enzyme purity was checked with sodium dodecyl sulfate polyacrylamide gel electrophoresis (SDS-PAGE). 10% polyacrylamide slab gels were used and the proteins were stained with Coomassie Brilliant Blue R-250. 0.5 μg of purified *N*-terminal variants was loaded per lane. As a molecular weight marker, Precision Plus Protein Standard (Biorad, Hercules, CA, USA) was used.

### 4.4. Analytical Gel Filtration

The hydrodynamic properties of the MBP-TtProDH variants were analyzed by size exclusion chromatography, as described previously [19]. In addition, 20 µM EE and 20 µM ΔABC in 50 mM sodium phosphate pH 7.4 were mixed, incubated at room temperature overnight, and subsequently analyzed by size exclusion chromatography.

### 4.5. ESI-MS

The native and denatured masses of the MBP-TtProDH variants were determined using nanoflow electrospray ionization mass spectrometry (ESI-MS) according to a previously established procedure [17,19], whereby the settings were optimized for the current application. Source backing pressure was increased to 7.8 mbar and the cone voltage was varied between 100 and 150 V.

### 4.6. Spectral Analysis

Far-UV circular dichroism (CD) spectra of the MBP-TtProDH variants were recorded, as described before [17]. 1 µM samples were prepared in 50 mM sodium phosphate, pH 7.4. Optical flavin absorption spectra of the MBP-TtProDH variants were recorded as has been described previously [19].

### 4.7. Enzyme Activity

Enzyme activity of the MBP-TtProDH variants was determined at 25 °C on a Hewlett Packard 8453 diode array spectrophotometer (Agilent Technologies, Santa Clara, CA, USA) using the proline:dichlorophenolindophenol (DCPIP) oxidoreductase assay [17]. For the standard assay, 0.07–0.66 of enzyme was added to a 600 µL reaction mixture containing 65 µM DCPIP and 100 mM l-proline in 50 mM sodium phosphate pH 7.4. Steady-state kinetic parameters were determined at 25 °C, essentially as described previously [19].

Proline oxidase activity of the MBP-TtProDH variants was determined at 25 °C in air-saturated 50 mM sodium phosphate pH 7.4, containing 3.5–7.5 µM enzyme, using a Hansatech Oxytherm system (Hansatech Instruments, King’s Lynn, UK). Reactions were started by the addition of 100 mM l-proline.

### 4.8. Inactivation with N-propargylglycine

The synthesis of *N*-propargylglycine was based on a procedure described before [23]. A clear solution of iodoacetic acid (1.05 g, 5.6 mmol) and *N*-propargylamine (2.6 g, 47 mmol) was refluxed in 50 mL aqueous ethanol for 24 h. The dark mixture was cooled to room temperature and the solvent was removed in vacuo. The crude product was precipitated from 1:1 ethanol:ethyl acetate. Recrystallization from aqueous ethanol and ethyl acetate and final drying under high vacuum yielded the *N*-propargylglycine as a white solid (28 mg, 4%). ^1^H-NMR (DMSO-d_6_): δ = 3.42 (d, 2H, *J* = 2.4 Hz), 3.24 (s, 2H), 3.16 (t, 1H, *J* = 2.4 Hz).

Spectral changes that were associated with the reaction of TtProDH with *N*-propargylglycine were monitored at 25 °C on a Hewlett Packard 8453 diode array spectrophotometer. A fresh stock solution of 75 mM *N*-propargylglycine was prepared in 50 mM sodium phosphate pH 7.4. 40 µM of the MBP-TtProDH variants in 50 mM sodium phosphate pH 7.4 was incubated with a final concentration of 2.5 mM *N*-propargylglycine. Immediately after the addition of *N*-propargylglycine, the first spectrum was recorded. Subsequent spectra were recorded at 1 min intervals for 90 min. Before and after the incubation of the enzyme variants with *N*-propargylglycine, 10 µL aliquots were removed from the cuvette and the enzyme activity was determined with the standard assay. Activity measurements were performed in triplicate.

## Figures and Tables

**Table 1:** Molecular masses of EE, ΔA, ΔAB, and ΔABC as determined by native and denatured ESI-MS. Both predicted (Pred.) as well as experimental (Exp.) masses are given in kDa with experimental errors less than 0.01%. For calculation of the predicted native masses, it has been taken into account that each subunit contains a non-covalently bound FAD cofactor (molecular mass 786 Da).

	Native						Denatured	
	Tetramer		Dimer		Monomer			
	Pred.	Exp.	Pred.	Exp.	Pred.	Exp.	Pred.	Exp.
MBP-TtProDH EE *	317.8	319.6	158.9	158.9	79.5	-	78.7	78.7
**MBP-TtProDH ΔA**	310.1	311.3	155.0	154.9–155.7	77.5	-	76.7	76.8
**MBP-TtProDH ΔAB**	305.1	307.6–307.8	152.6	152.6–153.7	76.3	-	75.5	75.5
**MBP-TtProDH ΔABC**	300.7	290.0–290.7	150.4	145.0–146.0	75.2	71.9–72.7	74.4	71.9
**MBP-TtProDH ΔAB V32D**	305.2	296.2–304.4	152.6	146.5–149.0	76.3	73.0–76.0	75.5	73.0 75.5
**MBP-TtProDH ΔAB Y35F**	305.1	307.4	152.5	152.3–152.9	76.3	-	75.5	75.5
**MBP-TtProDH ΔAB V36D**	305.2	296.2	152.6	151.1–151.9	76.3	-	75.5	73.0 75.5

* As determined previously [19].

**Table 2:** Kinetic parameters of the MBP-TtProDH variants at 25 °C, pH 7.4, as determined with the Proline:DCPIP oxidoreductase assay, using proline as the variable substrate, and specific activities of the MBP-TtProDH variants as determined by the proline:O_2_ assay, at a concentration of 100 mM proline. Each data point for the Proline:DCPIP assay was retrieved in triplicate; the data points for the Proline:O_2_ assay were retrieved in duplicate. The raw kinetic data of the Proline:DCPIP assay, used to determine the *K*_m_ and *k*_cat_, is provided in Table S1.

	Proline:DCPIP Assay			Proline:O_2_ Assay
	*K*_m_ (mM)	*k*_cat_ (s^−1^)	*k*_cat_/*K*_m_ (s^−1^ M^−1^)	Specific Activity (mU/mg)
MBP-TtProDH EE *	68 ± 8	9.8 ± 0.5	146	266 ± 12
**MBP-TtProDH ΔA**	116 ± 5	12.6 ± 0.3	109	405 ± 15
**MBP-TtProDH ΔAB**	60 ± 3	13.6 ± 0.3	229	228 ± 8
**MBP-TtProDH ΔABC**	114 ± 8	0.7 ± 0.02	6	20 ± 1
**MBP-TtProDH ΔAB V32D**	309 ± 25	3.2 ± 0.2	10	265 ± 4
**MBP-TtProDH ΔAB Y35F**	161 ± 29	14.1 ± 1.5	88	388 ± 5
**MBP-TtProDH ΔAB V36D**	189 ± 27	0.3 ± 0.03	1.6	21 ± 1

* As determined previously [19].

**Table 3:** Oligonucleotides used for the construction of the various MBP-TtProDH variants. For the helix αC variants, codon changes are underlined.

Variant	Oligonucleotide Sequence (5′ to 3′)
ΔA, forward	AATTAGAATTCCAGGTTGAACGTCTGATTAAACATCGTGCAAAAGG
ΔAB, forward	AAT TAGAATTCAAAGGTCTGGTTCGTCGTTATGTTGCCGGTG
ΔABC, forward	AATTAGAATTCCAGGTTGAACGTCTGATTAAACATCGTGCAAAAGG
ΔA, ΔAB, ΔABC, reverse	GCCCAAGCTTTTATTCTAGACCGCTAACCAGGC
ΔAB, V32D, forward *	CGAGGGAAGGATTTCAGAATTCAAAGGTCTGGATCGTCGTTATGTTGCCGGTGAAACCCTGG
ΔAB, Y35F, forward *	CGAGGGAAGGATTTCAGAATTCAAAGGTCTGGTTCGTCGTTTTGTTGCCGGTGAAACCCTGG
ΔAB, V36D, forward *	CGAGGGAAGGATTTCAGAATTCAAAGGTCTGGTTCGTCGTTATGATGCCGGTGAAACCCTGG

* As a reverse primer, the complement reverse sequence of the forward primer was used.

## Supplementary Materials

The following are available, Table S1: Raw kinetic data of the Proline:DCPIP assay of EE, ΔA, ΔAB, ΔABC and helix αC variants V32D, Y35F and V36D. Figure S1: Alignment of TtProDH, DrProDH, and the ProDH domains of BjPutA and GsPutA.

## Abbreviations

CD	circular dichroism
DCPIP	dichlorophenolindophenol
ESI-MS	electron spray ionization mass spectrometry
FAD	flavin adenine dinucleotide
FMN	flavin mononucleotide
GSA	glutamic semialdehyde
MBP	maltose-binding protein
ProDH	proline dehydrogenase
P5C	Δ^1^-pyrroline-5-carboxylate
P5CDH	Δ^1^-pyrroline-5-carboxylate dehydrogenase
PutA	proline utilization A
SDS-PAGE	sodium dodecyl sulfate polyacrylamide gel electrophoresis
Tt	*Thermus thermophilus*
EE	MBP-TtProDH with Phe10 and Leu12 replaced by Glu
ΔA	MBP-TtProDH lacking helix αA
ΔAB	MBP-TtProDH lacking helices αA and αB
ΔABC	MBP-TtProDH lacking helices αA, αB and αC
