# Supplementary material for: Functional Impact of the N-terminal Arm of Proline Dehydrogenase from Thermus thermophilus
Source: Molecules. 2018 Jan 16;23(1):184. doi: 10.3390/molecules23010184 (PMC6017737; doi:10.3390/molecules23010184)
Supplement: Supplementary file 1 [file molecules-23-00184-s001.pdf]

Supplementary Material

Functional Impact of the N-terminal Arm of Proline Dehydrogenase from *Thermus thermophilus*

Mieke M.E. Huijbers [1], Ilona van Alen [1], Jenny W. Wu [1], Arjan Barendregt [2,3], Albert J.R. Heck [2,3], and Willem J.H. van Berkel [1,*]

[1] Laboratory of Biochemistry, Wageningen University & Research, Stippeneng 4, 6708 WE Wageningen, The Netherlands; willem.vanberkel@wur.nl

[2] Biomolecular Mass Spectrometry and Proteomics, Bijvoet Center for Biomolecular Research and Utrecht Institute of Pharmaceutical Sciences, Utrecht University, Padualaan 8, 3584 Utrecht, The Netherlands; a.j.r.heck@uu.nl

[3] Netherlands Proteomics Center, Padualaan 8, 3584 Utrecht, The Netherlands; a.jr.heck@uu.nl

* Correspondence: willem.vanberkel@wur.nl; Tel.: +31-6-120-77313

Table S1. Raw kinetic data of the Proline:DCPIP assay of EE, ΔA, ΔAB, ΔABC and helix αC variants V32D, Y35F and V36D. Each data point provided here is the average of three measurements. This kinetic data was used to determine the kinetic parameters K_m and k_{cat}, which are provided in Table 2.

	**EE**	**ΔA**	**ΔAB**	**ΔABC**
Proline (mM)	k_{obs} (s^{-1})	k_{obs} (s^{-1})	k_{obs} (s^{-1})	k_{obs} (s^{-1})
0.5	0.07 ± 0.002	0.08 ± 0.018	0.14 ± 0.005	0.00 ± 0.000
2.5	0.33 ± 0.002	0.29 ± 0.012	0.64 ± 0.008	0.00 ± 0.000
5	0.65 ± 0.015	0.56 ± 0.016	1.16 ± 0.020	0.03 ± 0.002
10	1.36 ± 0.069	1.08 ± 0.000	2.06 ± 0.069	0.06 ± 0.002
20	2.11 ± 0.085	1.90 ± 0.044	3.53 ± 0.060	0.11 ± 0.009
35	3.01 ± 0.129	2.91 ± 0.038	4.90 ± 0.066	0.15 ± 0.004
50	4.06 ± 0.262	3.89 ± 0.008	6.20 ± 0.307	0.20 ± 0.001
75	5.54 ± 0.242	4.94 ± 0.043	7.59 ± 0.027	0.25 ± 0.009
100	6.22 ± 0.357	5.66 ± 0.100	8.38 ± 0.234	0.30 ± 0.014
150	6.89 ± 0.445	7.06 ± 0.274	10.09 ± 0.573	0.37 ± 0.011
200	7.00 ± 0.406	8.08 ± 0.121	10.38 ± 0.219	0.42 ± 0.010

	**V32D**	**Y35F**	**V36D**
Proline (mM)	k_{obs} (s^{-1})	k_{obs} (s^{-1})	k_{obs} (s^{-1})
0.5	0.01 ± 0.003	0.09 ± 0.009	0.00 ± 0.001
2.5	0.03 ± 0.005	0.35 ± 0.019	0.01 ± 0.002
5	0.05 ± 0.003	0.66 ± 0.017	0.02 ± 0.002
10	0.10 ± 0.010	1.16 ± 0.004	0.02 ± 0.003
20	0.20 ± 0.008	1.88 ± 0.183	0.03 ± 0.003
35	0.33 ± 0.009	2.82 ± 0.087	0.03 ± 0.005
50	0.45 ± 0.009	3.34 ± 0.137	0.04 ± 0.002
75	0.63 ± 0.020	3.95 ± 0.257	0.04 ± 0.003
100	0.75 ± 0.002	5.14 ± 0.489	0.05 ± 0.002
150	1.01 ± 0.042	6.94 ± 0.836	0.06 ± 0.001
200	1.26 ± 0.029	7.93 ± 0.445	0.07 ± 0.001

```
TtProDH         MNLDLAYRSFVLGVAGHPQVERLIKHRAKGLVRRYVAGETLEEALKAAEALEREGVHAIL
DrProDH         -MIDQLYRKAVLTVAERPQVEQLARQKMWNLAERFVAGESIESAIQAVQALERDGIAGNL
BjPutA          ---------------------------------QFVLGEDIRAAMKRASGMEQKGYTYSY
GsPutA          ------------------------------------VGETTKEAVKNLEKLRKDGFAAVV
                                                     **  . *::  . :.:.*

TtProDH         DLLGEMVRTEEEARAFQRGLLELVWALAGKP----------WPKYI----------SLKL
DrProDH         DLLGEFIDSPAKCTEFADDVIKLIEAAHAAG----------IKPYV----------SIKL
BjPutA          DMLGEAARTDADAMRYHAAYGRAIEAIAKGCGSEDVR----RNPGISV----------KL
GsPutA          DVLGEATLSEEEAEVYTNTYLELLEALKKEQGSWKGLPGKGGDPGLDWGHAPKVNIAVKP
                *:***   :  ..  :     . : *                   :            *

TtProDH         TQLGLDL-------SEDLALALLREVLREAEPRGVFVRLDMEDSPRVEATLRLYRALR--
DrProDH         SSVGQGKD----ENGEDLGLTNARRIIAKAKEYGGFICLDMEDHTRVDVTLEQFRTLVG-
BjPutA          SALHPRYEEAQKDRVTAELYPRLRDLALMARGRRMGLNIDAEEADRLSISMDLIERLMRD
GsPutA          TALFCLANPQDFEGSVVAILDRMRRIFKKVMELNGFLCIDMESYRHKEIILEVFRRL--K
                : :                    * :   .      : :* *.  : .  :   . *

TtProDH         --EEGFSQVGIVLQSYLYRTEKDLLDLLP------YRPNLRLVKGAYREPKEVA------
DrProDH         --EFGAEHVGTVLQSYLYRSLGDRASLDD------LRPNIRMVKGAYLEPATVA------
BjPutA          PGLAGWDGLGVVVQAFGQRCGAVLEYLHALAEETDRRIMVRLVKGAYWDTEIKLAQVEGL
GsPutA          LEYRDYPHLGIVLQAYLKDNDKDLDDLLAWAKEHKVQISVRLVKGAYWDYETVKAKQN-D
                    .   :* *:*::          *         :  :*:***** :

TtProDH         -----FPDKRLIDAEYLHLGK--LALKEGLYVAFATHDPRIIAELKRYTEAMGIPRSRFE
DrProDH         -----YPDKADVDQNYRRLVF--QHLKAGNYTNVATHDERIIDDVKRFVLAHGIGKDAFE
BjPutA          AGFPVFTSKPATDVSYIANARRLLNMTDRIYPQFATHNAHTVAAILDMAE----DKGSFE
GsPutA          WEVPVWTIKAESDAAYERQARKILENHQICHFACASHNIRTISAVMEMARELNVPEDRYE
                     :  *   *  *              :   *:*: : :  :   .      .. :*

TtProDH         FQFLYGVRPEEQR-RLAREGYTVRAYVPYGR--DWYPYLTRRIAERPENLLLVLRSLVSG
DrProDH         FQMLYGIRRDLQK-QLAAEGYRVRVYLPYGR--DWYAYFSRRIAETPRNAAFVVQGMLKG
BjPutA          FQRLHGMGEALHDIVLKSEGTRCRIYAPVGAHKDLLAYLVRRLLE---------------
GsPutA          FQVLYGMAEPVRKGILKVAG-RIRLYAPYGNMVPGMGYLVRRLLENTANE----------
                ** *:*:    :   *   *   * * * *       *: **: *
```

Figure S1. Sequence alignment of TtProDH, DrProDH, and the ProDH domains of BjPutA and GsPutA. The alignment was generated using Clustal Omega [1, 2]. The protein sequences were retrieved from the UniProt Knowlegdebase (UniProtKB). Sequence accession numbers: TtProDH: Q72IB8, DrProDH: Q9RW55, BjPutA: A0A0H4LBV6, GsPutA: Q746X3. For BjPutA and GsPutA, only the sequence comprising the ProDH domain was used (BjPutA: residues 172 – 465 [3], GsPutA: residues 121 – 430 [4]). In green, the conserved Arg-Glu ion pair (Arg288-Glu65 in TtProDH), which is suggested to act as an active site gate, is shown.

References

1. Goujon, M.; McWilliam, H.; Li, W.; Valentin, F.; Squizzato, S.; Paern, J.; Lopez, R. A new bioinformatics analysis tools framework at EMBL–EBI. *Nucleic Acids Res.* **2010**, *38*, W695-W699.
2. Sievers, F.; Wilm, A.; Dineen, D.; Gibson, T.J.; Karplus, K.; Li, W.; Lopez, R.; McWilliam, H.; Remmert, M.; Söding, J., et al. Fast, scalable generation of high-quality protein multiple sequence alignments using Clustal Omega. *Mol. Syst. Biol.* **2011**, *7*, 1-6.
3. Srivastava, D.; Schuermann, J.P.; White, T.A.; Krishnan, N.; Sanyal, N.; Hura, G.L.; Tan, A.; Henzl, M.T.; Becker, D.F.; Tanner, J.J. Crystal structure of the bifunctional proline utilization A flavoenzyme from *Bradyrhizobium japonicum*. *Proc. Natl. Acad. Sci. USA* **2010**, *107*, 2878-2883.
4. Singh, H.; Arentson, B.W.; Becker, D.F.; Tanner, J.J. Structures of the PutA peripheral membrane flavoenzyme reveal a dynamic substrate-channeling tunnel and the quinone-binding site. *Proc. Natl. Acad. Sci. USA* **2014**, *111*, 3389-3394.
